# Examining Emailed Feedback as Boosters After a College Drinking Intervention Among Fraternities and Sororities: Rationale and Protocol for a Remote Controlled Trial (Project Greek)

**DOI:** 10.2196/42535

**Published:** 2022-10-28

**Authors:** Abby L Braitman, Jennifer L Shipley, Megan Strowger, Rachel Ayala Guzman, Alina Whiteside, Adrian J Bravo, Kate B Carey

**Affiliations:** 1 Department of Psychology Old Dominion University Norfolk, VA United States; 2 Union College Schenectady, NY United States; 3 Department of Psychological Sciences William & Mary Williamsburg, VA United States; 4 Department of Behavioral and Social Sciences Center for Alcohol and Addiction Studies Brown University School of Public Health Providence, RI United States

**Keywords:** college drinking, fraternities, sororities, web-based intervention, boosters

## Abstract

**Background:**

College students involved in Greek life (ie, members of fraternities and sororities) tend to engage in more high-risk alcohol use and experience more negative consequences than those not involved in Greek life. Web-based alcohol interventions, such as Alcohol eCHECKUP TO GO, have been successful in reducing alcohol use and consequences among the general college student population, but interventions targeting alcohol reduction among those involved in Greek life have had limited success. Booster emails including personalized feedback regarding descriptive norms and protective behavioral strategies have shown potential in increasing the effectiveness of web-based interventions among college drinkers. Studies are needed to determine the efficacy of these boosters among those involved in Greek life.

**Objective:**

The primary objective of this study is to assess the efficacy of booster emails sent to Greek life students who complete Alcohol eCHECKUP TO GO. Specifically, we expect that participants who receive the booster emails will reduce their alcohol consumption and related problems (primary aim 1), reduce perceived peer drinking, and increase the number of protective behavioral strategies they use over time (primary aim 2) relative to those who do not receive boosters. Contingent upon finding the emailed booster efficacious and sufficient enrollment of members from each organization, an exploratory aim is to examine social mechanisms of change (ie, through selection vs socialization).

**Methods:**

This study is a remote, controlled intervention trial following participants for up to 6 months. Participants must be aged at least 18 years, undergraduate students, and members of a participating fraternity or sorority. Eligible participants complete a web-based baseline survey to assess their alcohol consumption behaviors and beliefs, including norms and protective behavioral strategies, and information about their social networks. After completing the baseline survey, they participate in the web-based intervention. Follow-up surveys are sent 1, 3, and 6 months after the intervention. Those in the booster condition also receive emails containing personalized feedback at 2 weeks and 14 weeks after the intervention. Latent growth models and R-Simulation Investigation for Empirical Network Analysis will be used to analyze the data.

**Results:**

As of September 2022, we have enrolled 18 participants from 2 fraternities and 2 sororities, and they have completed the baseline survey. Overall, 72% (13/18) of participants have completed the 1-month follow-up. Enrollment will continue through December 2022.

**Conclusions:**

This study aims to examine the effectiveness of personalized feedback booster emails sent after an alcohol intervention among members of college Greek life. A secondary, exploratory aim is to provide information about social mechanisms of change (if possible). The current methodology targets whole network recruitment, with chapter presidents serving as gatekeepers and facilitators. Unique challenges of recruiting whole networks and working with campus administrators are discussed.

**Trial Registration:**

ClinicalTrials.gov NCT05107284; https://clinicaltrials.gov/ct2/show/NCT05107284

**International Registered Report Identifier (IRRID):**

DERR1-10.2196/42535

## Introduction

### Background

College drinking is prevalent and linked to numerous academic problems and physical consequences [1-6]. Moreover, members of fraternities and sororities tend to drink more and do not respond well to interventions designed to reduce drinking or related harm, as described in the following sections. Boosters are a promising way to strengthen and extend the effects of web-based interventions, while maintaining low cost and easy dissemination. This study examines whether a personalized feedback booster delivered via email after a web-based intervention enhances outcomes relative to the intervention alone among members of fraternities and sororities.

### Drinking Among Members of Fraternities and Sororities

College students involved in Greek life (eg, members of fraternities or sororities) tend to consume more drinks [7-10] and report more negative alcohol-related consequences than non–Greek-involved students [7,9,11-13]. Not only do they consume more drinks but they are also more likely to engage in binge drinking [10-14] than their non-Greek counterparts. Rates of binge drinking among Greek-involved students range from 70.4% to 86% [13-15] and approximately two-thirds (64%) of members of fraternities engage in frequent binge drinking (defined as consuming ≥5 drinks on at least three occasions during the past 2 weeks) [15]. These high rates of heavy drinking suggest that students involved in Greek life represent a particularly high-risk group of drinkers.

Students involved in Greek life are highly socially connected, potentially influencing their heavy drinking behaviors. Previous studies have found that alcohol consumption is high among Greek-involved students who live in fraternity or sorority houses compared with those who are members but do not live in Greek housing [13,14,16], suggesting that fraternities and sororities are close-knit networks that tend to drink more together than apart. A longitudinal social network study of fraternity members revealed that members who “hang out” together tend to consume similar quantities of alcohol, and those who hang out with heavy drinkers tend to either drink heavily already or increase their alcohol consumption over time [17]. Moreover, a series of studies tracking drinking behaviors and Greek membership over time demonstrated that although heavy drinkers are more likely to join Greek organizations, there is also an immediate and sustained influence to increase and maintain heavy drinking over time via environmental influences [18,19]. Those who join Greek organizations demonstrate increased drinking, whereas those who disaffiliate demonstrate decreased drinking [18]. These findings suggest that the drinking behaviors of Greek-involved students, already an at-risk group for heavy drinking, are influenced by strong social connections with other Greek-involved students.

### Interventions Among Members of Fraternities and Sororities

Given that student members of fraternities and sororities consume more alcohol and report more consequences than nonmembers, they have been a target for alcohol harm reduction interventions. However, a meta-analysis of 21 different alcohol interventions with this population revealed few significant reductions in alcohol consumption or related problems after the intervention relative to controls [20]. Interventions yielded limited success, with some reductions observed in the number of drinks consumed on specific occasions or frequency of drinking (small to medium effect sizes). In addition, interventions that were brief (<60 minutes) versus long (>60 minutes) yielded strong reductions in heavy drinking frequency. The limited intervention success in this population coupled with strong effects of brief interventions suggest that this population may be an ideal target for personalized feedback booster emails after a brief web-based intervention.

### Alcohol eCHECKUP TO GO

Alcohol eCHECKUP TO GO is a brief web-based alcohol education program that asks questions about alcohol use, related perceptions, and risks and provides a personalized feedback report. It has strong empirical support and is listed as program of high effectiveness by the National Institute on Alcohol Abuse and Alcoholism College Alcohol Intervention Matrix [21]. The program has been shown to be efficacious in reducing alcohol use and related risks among incoming students [22-28] and samples of general college drinkers [29,30]. Reductions in alcohol use after administration of Alcohol eCHECKUP TO GO have also been demonstrated among specific high-risk college populations, such as heavy drinkers or frequent heavy drinkers [31-36], heavy or problematic drinkers [37,38], or students mandated to receive treatment [39-42]. Despite these strong successes with high-risk college populations, so far, no studies have examined the efficacy of Alcohol eCHECKUP TO GO specifically among students involved in Greek life.

### Boosters for Web-Based College Drinking Interventions

Boosters refer to brief or delayed maintenance sessions aimed at increasing an intervention’s efficacy [43]. Using boosters as a technique to supplement programs or interventions has been effective in a variety of fields for further promoting healthful behaviors or reducing problematic behaviors, such as by promoting physical activity [44], improving family functioning [45], and reducing harmful alcohol use [46]. Given that the effects of in-person college drinking interventions on peak drinking tend to decay by 27 weeks and that web-based college drinking intervention effects tend to decay by 14 weeks (refer to the paper by Carey et al [47] for a meta-analysis), it is important to use methods designed to increase the effects of college drinking interventions, such as boosters. Few studies have examined the long-term efficacy of boosters in reducing alcohol use among college students [48,49].

Emailed boosters (ie, follow-up emails after the intervention) [43,49] are an efficient and inexpensive delivery method. However, evidence is equivocal regarding the long-term efficacy of emailed boosters for web-based college drinking interventions [49,50]. The content of emailed boosters may prove to be key and may be informed by efficacious components of brief interventions. Effective methods of reducing college drinking include personalized normative feedback (ie, providing accurate information about peer drinking, often correcting normative misperceptions) [51] and promoting protective behavioral strategies (ie, engaging in techniques to reduce alcohol consumption, problems, or both) [52]. Importantly, despite the limited studies on boosters after college drinking, personalized normative feedback via boosters has been shown to prolong the effects of reduced drinking after the intervention [43,48,49,53]. In total, 2 studies using personalized feedback about both norms and protective behavioral strategies as content for emailed boosters observed a continuous reduction in reported alcohol use among college students at 4 weeks [43] and among legal drinking-aged college students for up to 9 months after the intervention [49], thus further demonstrating the potential effectiveness of emailed boosters in a college population.

### Normative Perceptions and Protective Behavioral Strategy Use in Greek Life

Both descriptive drinking norms and protective behavioral strategy use, key components in the booster feedback deployed as part of this study, have strong links to drinking among members of fraternities and sororities. Descriptive drinking norms have been found to mediate associations between involvement in Greek life and alcohol use [7], with members of Greek life holding higher normative perceptions and greater norms being linked to greater consumption. There is conflicting evidence regarding whether fraternity and sorority members use greater or fewer protective behavioral strategies than students not involved in Greek life. Barry et al [54] found that Greek-involved students used few strategies, whereas Soule, Barnett, and Moorhouse [12] found that they used more. Regardless of whether members of Greek life use few versus many of these strategies, Barry et al [54] found when controlling for alcohol consumption, that using more protective behavioral strategies was linked to reporting fewer alcohol-related problems among fraternity and sorority members, suggesting that they are key to reducing harm. Taken together, personalized feedback regarding drinking norms and protective behavioral strategy use may be useful to be included in emailed boosters after college drinking interventions delivered to students involved in Greek life.

### Study Objectives

#### Overview

Members of sororities and fraternities engage in heavier drinking than their non-Greek counterparts and often report more consequences [7,9,11-13]; furthermore, previous intervention efforts with this population have yielded limited success [20]. Therefore, this study aims to strengthen and extend alcohol intervention effects by using personalized feedback boosters sent via email. Moreover, given the close connections among members of fraternities and sororities, these may be closed peer networks that can facilitate the examination of how changes in drinking occur through social influence. In other words, we can potentially examine if change happens through *selection* (ie, transitioning into friendships with individuals who are similar), such that those who drink less after the intervention change who they drink with to peers who also drink less, or *socialization* (ie, becoming more similar to individuals who one spends time with), such that participants may drink less if their drinking buddies are drinking less after the intervention and booster. Thus, this study has 2 primary aims (to be examined regardless of study outcomes or participation rates) and 1 exploratory secondary aim (to be examined only if the booster is efficacious and most members of each enrolled organization complete the study).

#### Primary Aim 1

We will examine the efficacy of a personalized feedback booster emailed after Alcohol eCHECKUP TO GO is delivered to members of fraternities and sororities. We hypothesize initial postintervention drinking reductions for both study conditions, with individuals in the booster condition reporting further reductions at later follow-ups.

#### Primary Aim 2

Given the focus on perceived descriptive norms and protective behavioral strategies in the personalized feedback boosters, we will examine whether the booster affects changes in those constructs over time. We hypothesize that individuals in the booster condition will report further reductions in norms and increases in protective behavioral strategies at later follow-ups.

#### Exploratory Aim

If the emailed booster is efficacious and most members of each enrolled organization complete the study, we will examine social mechanisms of change (ie, through selection vs socialization).

In the following sections, we review the methodological approach currently used, challenges (eg, administrative red tape, gatekeepers in recruitment, change in health programing, and extended time lines), and protocol revisions executed in response to these challenges (eg, changing data collection sites and seeking additional approvals).

## Methods

### Project Overview and Design

Project Greek is an ongoing clinical trial of a personalized feedback booster emailed to members of fraternities and sororities after they complete Alcohol eCHECKUP TO GO, a web-based intervention designed to reduce college drinking. Study conditions include *intervention only* versus *intervention plus booster*. Participants are assigned to a condition at the organization level so that all members of an organization are in the same condition (ie, all enrolled members of the organization received a feedback booster email or all enrolled members of the organization did not). In both conditions, participants attend a virtual baseline session where they complete a survey and Alcohol eCHECKUP TO GO and are invited to complete follow-up surveys 1, 3, and 6 months later. Tailored feedback booster emails are sent 2 weeks after the intervention (known to be effective) [43,49] and 14 weeks after (known to be the window when web-based intervention effects wane based on meta-analysis) [47]. At each time point, participants complete a survey about their current alcohol-related behaviors, cognitions, and beliefs. They are also asked to name close network members at each time point to allow exploration of the influence of socialization versus selection via social network analysis.

### Setting and Participant Selection

Eligibility criteria include being aged ≥18 years, an undergraduate student, and a member of a participating fraternity or sorority. Alcohol criteria were omitted because (1) we want to enroll as many members of participating organizations (fraternities and sororities) as possible to facilitate the social network examination involved in the secondary aim of the study and (2) given rates of alcohol use among members of Greek life (described previously), this seemed unnecessary.

Data collection was originally planned for the research team’s host institution, a minority serving, large, public institution. It is a majority commuter campus, with only 20.1% of students living on campus in spring of 2019 (before the COVID-19 global pandemic). Moreover, 25% of students are affiliated with the military (including spouses and children of those active in the military). After forming a student advisory panel comprising undergraduate students in sororities or fraternities and consulting them on the study design, this plan was changed. We learned that many members of fraternities and sororities at this institution do not consider other members of the same organization to be close friends, and they often drink alcohol with individuals who are not in these organizations. If asked to list their top 5 closest friends, most of them would not include members of their Greek organization. This is because many students maintain connections with peers from before joining the institution (eg, they still live near childhood friends) and many have responsibilities outside college (eg, taking care of family or working at a job). This would be problematic for the secondary aim of the study, as we hoped to recruit closed social networks of drinkers by recruiting all members of a participating fraternity or sorority. Given how many close friends and drinking buddies are not in their social organization, recruiting through fraternities and sororities at the host institution would likely be insufficient for recruiting participants’ close friends and drinking buddies.

Data collection now occurs at a nearby institution. Both schools are public, 4-year institutions that also offer advanced degrees. However, the new data collection site is a medium-sized university with a requirement that full-time students must live on campus during their first 2 years. This facilitates creating new social ties with fellow students at the institution over maintaining old ties with friends from high school. Moreover, there is a strong presence of Greek life, with 32% of undergraduate men and 36% of undergraduate women involved in fraternities or sororities and with 13 sorority and 16 fraternity chapters on campus. These conditions are more favorable for recruiting closed networks of drinkers via fraternities and sororities.

The student advisory panel provided suggestions for incentives, one of which was donating to the organization’s charity of choice or philanthropy if a specific threshold of members of the organization participated in the study. However, this student-generated option was not approved by the institutional review board (IRB), with concerns that this form of incentive could lead to peer pressure to participate. As such, all incentives for participation are individual in nature. Participants receive a gift card worth US $20 for their baseline participation and a promotional item with their organization’s Greek letters or crest on it (eg, sticker or keychain). Participants receive US $5 for each completed follow-up survey (for the 1-, 3-, and 6-month assessments). As an additional incentive, participants who complete all assessments will be given US $5 as bonus (yielding US $40 in total if all follow-ups are completed). All individual monetary compensation is provided via web-based gift card.

### Ethics Approval

The study protocol was approved by the Old Dominion University IRB (protocol 1565916-7). As noted previously, approval was also secured by the William & Mary Protection of Human Subjects Committee (protocol PHSC-2021-12-31-15372) and the National Panhellenic Conference’s research committee.

### Study Procedures

#### Overview

We worked with the school administrators and chapter presidents to obtain member lists and recruit potential participants. Participants may schedule their baseline session at a time of their choice (completely web-based) and meet with a research assistant via web-based meeting platform. Follow-up emails are sent 1, 3, and 6 months after participation, with daily reminders sent for up to 30 days (or until the relevant survey is completed). We ask participants to opt in to alternative contact methods in the baseline assessment (ie, nonschool email addresses and phone numbers for texting) to facilitate high retention in the follow-up surveys. This study is registered in ClinicalTrials.gov (NCT05107284) and is funded by the National Institute on Alcohol Abuse and Alcoholism (refer to Multimedia Appendix 1 for the summary statement provided during grant peer review).

#### Recruitment

To obtain member lists for recruitment purposes, we asked the campus administrators to share information about who is involved in Greek life. Although they were amenable to this request, they required more protection than that required by the federal regulations or IRB. For instance, they required that we secure approval from the research committee of the National Panhellenic Conference (a national network with 26 sororities). Campus administrators also requested that the recruitment site’s IRB review the proposal, even though we had an institutional agreement naming the host institution as the lead approving IRB. Approval was eventually obtained from the National Panhellenic Conference’s research committee and the site IRB, but this created some project delays. After securing all relevant approvals, the campus administrators provided the names and institutional email addresses for presidents of local chapters of the 16 fraternities and 13 sororities.

We grouped chapters with similar membership sizes, and for our first round of selection, we randomly assigned one from each pair to each study condition. We will continue selecting and enrolling chapters until the target enrollment is reached. After selecting a round of chapters, we contacted the presidents of those chapters to explain the purpose and design of the study, share our approvals from the relevant IRBs and the National Panhellenic Conference’s research committee, and share that we have a Certificate of Confidentiality from the National Institutes of Health. Presidents who opt in for their chapter’s participation share membership lists (names and email addresses), help in selecting a promotional item for compensation purposes, and help in identifying optimal data collection windows (described in more detail in the Baseline section). Names on membership lists are used for social network assessment in each survey (described in more detail in the Measures section). Emails are sent to all members in the participating organizations. This email contains content similar to that of the email sent to chapter presidents (ie, study purpose and design, relevant approvals, and compensation structure). We also ask chapter presidents if we may attend a chapter meeting to describe the study in more detail and answer questions. Overall, 25% (1/4) of the enrolled organizations have chosen to do this so far. Undergraduate research assistants, who are also members of Greek life, make these visits. Recruitment emails include a link to schedule a web-based baseline session.

#### Baseline

The project staff work with each chapter president to identify times that may work well for most members (such as participating before or after a chapter meeting), but several other meeting times are also available to maximize the availability of participation. Participants schedule their participation time through a web-based time management system. They receive an email reminder from the time management system 24 hours before their assigned time slot. Regardless of the condition, participants complete a baseline session with research staff over a meeting platform, with cameras and microphones enabled. Upon signing in to the meeting, the research assistants provide participants with a link (using the chat function) to a web-based survey. Participants read and view videos of the study procedures on the first page of this website. After reviewing the videos, participants are provided with the informed consent document and have the opportunity to ask questions. After consenting to participate, the web page automatically loads the baseline survey (approximately 30-45 minutes), which assesses their behaviors over the past 30 days.

After completing the initial assessment, participants are directed to navigate through a web-based intervention program to address college drinking (Alcohol eCHECKUP TO GO) until it is completed (approximately 20-30 minutes). As participants navigate through the website, research assistants remain on the virtual meeting to ensure that the participants do not go off-task such as walking away from the computer screen or looking at their phone. However, they do not monitor the participants’ progress or responses through the program. Baseline sessions can accommodate up to 20 participants per session, but typically, 1-2 participants participate per session.

#### Intervention

When we first contacted the campus administrators at the data collection institution, all incoming students were required to complete alcohol programing before arriving on campus for their first semester, specifically AlcoholEDU for College. This program has been empirically supported, with a recent meta-analysis revealing consistent reductions in overall and peak drinking after the intervention [55]. However, the institution changed midyear to another program that lacks empirical support. Moreover, after examining the content of the new program, we saw an absence of empirically supported components such as personalized normative feedback, an important component for effective interventions [51,56]. Although there is exposure to an alcohol education program before matriculation (either AlcoholEDU for College or another), Alcohol eCHECKUP TO GO is different, and therefore offers new information to Greek life participants.

Alcohol eCHECKUP TO GO is a web-based intervention customized to each institution using it, such as including local resources and institution-specific norms. The program asks students questions (such as expectations about drinking alcohol, individual risk factors, perceptions of peer use, student goals, etc), and then provides a personalized feedback report. Alcohol eCHECKUP TO GO has been repeatedly documented as efficacious, with several studies documenting its efficacy among college students, as noted in the Introduction section. It was tailored for this study, with normative information specific to the data collection institution.

#### Follow-up Sessions

Approximately 1, 3, and 6 months after the initial assessment, researchers send each participant an email inviting them to complete a follow-up assessment, which contains a link to the web-based survey. Daily reminders are sent for up to 30 days or until the relevant survey is completed. If participants opt in to provide additional ways to contact them (a second email address or a phone number to send SMS text message), these methods are also used to send the link to the follow-up surveys.

#### Booster

Personalized feedback emailed to participants at 2 and 14 weeks after the baseline session serve as a booster to the original intervention. Baseline data are used to provide students with normative information and reminders of protective behavioral strategies they can use to reduce drinking-related harm. The personalized normative feedback uses institution-specific data from previous studies by the research team. The feedback compares (1) their typical weekly consumption provided at baseline, (2) their normative perceptions (ie, what they think their close friends and other typical students at their university consume), (3) the average consumption of actual male and female students at their university, and (4) the percentage of gender-matched students at their university who drink less than them. This information is provided to participants with a colorful bar graph and accompanying text. The reminders of harm reduction strategies (eg, “Alternating alcoholic and nonalcoholic beverages when you are drinking”) are presented separately for strategies participants report using versus strategies they may consider starting to use. A tracking image is included to record if and when each booster email is viewed.

### Measures

#### Overview

All participants complete a computerized survey at the beginning of their baseline session that assesses alcohol use, alcohol-related problems, protective behavioral strategies for drinking and their perceived effectiveness, alcohol-related cognitions (motives, expectancies, and beliefs about alcohol use), cannabis and tobacco use, COVID-19 pandemic experiences, internalizing symptoms (symptoms of stress, anxiety, and depression), demographics, and social network (refer to Table 1 for list of all measures and citations). The social network assessment can be operationalized into network-level variables (eg, proportion of heavy drinkers in their network), but participants report who specifically they are friends with and whom they drink with, which will allow for the examination of the exploratory secondary aim about selection versus socialization. The primary outcomes of the study are alcohol use and related problems, as these are expected to reduce after the intervention and booster. Secondary outcomes include normative perceptions and protective behavioral strategies, given that they are directly addressed by the personalized booster feedback. Other constructs assessed are potential moderators (eg, alcohol-related cognitions and internalizing symptoms) or covariates (eg, pandemic experiences and demographics). Most constructs are assessed at all time points, but some are assessed only during the baseline session. Table 1 contains a complete list of the constructs assessed, measures used, and time points assessed for each.

**Table 1:** Constructs assessed in the study, including measure used and time points assessed.

Construct		Description	Measure	Time points assessed
**Primary outcomes**				
	Alcohol use	Number of drinks consumed each day of a typical week for the past 30 days and number of hours lapsed while drinking	Daily Drinking Questionnaire [57]	All
	Alcohol-related problems	Problems that participants report related to their alcohol use for the past 30 days	Brief Young Adult Alcohol Consequences Questionnaire [58]	All
**Secondary outcomes**				
	Protective behavioral strategies	Unidimensional measure of strategies one can use to reduce drinking and related harms (past 30 days)	Protective Drinking Practices Scale [59]	All
	Normative perceptions of peer drinking	Descriptive norms (perceptions of how much close friends and peers at the same institution drink) and injunctive norms (perceptions of close friends’ approval of drinking)	Injunctive norms adapted from the study by Carey et al [60]	All
	Social network	Assessment of behaviors of close friends in their organization and out of their organization (eg, drinking and social behaviors)	Adapted version [61] of the Brief Important People Interview [62]	All
**Other measures**				
	Alcohol expectancies	Expectations about the effects of alcohol on an individual	Comprehensive Effects of Alcohol Questionnaire [63]	Baseline
	Drinking motives	Why one engages in alcohol use	Drinking Motives Questionnaire–Revised [64]	Baseline
	Alcohol beliefs	How salient alcohol use is to college life	College Life Alcohol Salience Scale [65]	Baseline
	Cannabis and tobacco use	Current, past month, and lifetime use	Created by the researchers	All
	Cannabis beliefs	How salient cannabis use is to college life	Perceived Importance of Marijuana to the College Experience Scale [66]	Baseline
	Pandemic experiences	Questions about participants’ experiences with the COVID-19 pandemic (eg, stressors)	Created by the researchers	Baseline
	Depression	Measure of symptoms of depression in the past 30 days	Center for Epidemiologic Studies Depression Scale-10 [67]	Baseline
	Anxiety	Measure of symptoms of general anxiety in the past 30 days	General Anxiety Disorder-7 [68]	Baseline
	Stress	Measure of symptoms of perceived stress in the past 30 days (specifically the vulnerability subscale)	Perceived Stress Scale–Revised [69]	Baseline
	Demographics	Information such as age, race, sex, GPA^a^, class standing, athletic status, student status (full-time student vs part-time student), residential status, relationship status, and sexual identity	N/A^b^	Baseline

^a^GPA: grade point average.

^b^N/A: not applicable.

#### Attention Checks

Given the length of the surveys, each assessment contains several attention checks. These are items that are either directive in nature (eg, “For this item, select ‘most of the time’”) or have a clearly correct answer (eg, “Which is the highest number?”). They are included to detect inattention, such as if a participant is clicking through the survey without fully reading the items. Participants who are not fully providing their attention to the survey can introduce noise in the data and attenuate the study’s power [70]. Given the investment in each participant in a study with this design (a longitudinal assessment of select individuals within specifically targeted organizations), it is not beneficial to exclude the data of inattentive individuals. Instead, live feedback is provided to direct them to focus their attention: “Your answer for this question is not correct. Your responses are very important to us. Please be sure to read questions thoroughly and answer carefully.” Then, participants have to select the correct answer before moving on to the next question.

### Data Analysis Plan

Before hypothesis testing, the data will be examined for normality and outliers. Histograms and values for skewness and kurtosis will be examined. Positively skewed variables will be natural log transformed, unless paired with an excessive number of zeroes. If there are an excess of zeroes in an outcome, the variable will be dichotomized if other values are not well represented or appropriate modeling techniques will be used (eg, hurdle models) if other values are well represented. Boxplots and IQRs will be used to check for outliers. Extreme values will be winsorized (ie, cases are retained in the sample, but values are made less extreme). Cases with missing data will be compared with complete cases across major study variables to identify whether there are systematic differences in missingness. If significant associations are identified, these variables will be used as covariates in later analyses.

For primary aim 1, latent growth models will be used to examine the efficacy of the personalized feedback boosters sent via email. The model will specify 1 intercept and 2 slopes to capture initial postintervention change (with slope 1 loadings coded as 0 for baseline and 1 for each follow-up assessment) versus long-term impacts on alcohol use (slope 2 loadings coded as 0, 0, 2, and 5 to reflect months since the first follow-up). Multiple models will be conducted specifying different forms of the second slope (eg, linear vs quadratic change), with the best-fitting model serving as the final model. The intercept and both slopes will be regressed on study condition, with booster efficacy demonstrated as a significant, negative coefficient for the impact of study condition on slope 2. Separate models will be conducted for alcohol use versus alcohol-related problems (with time-varying covariates for alcohol use; eg, problems at month 1 will control for alcohol quantity at month 1). Sex will be controlled for in all models.

Given that the tailored feedback boosters address both descriptive drinking norms and protective behavioral strategies, these are considered as secondary outcomes. Models identical to the one described previously will be conducted (eg, latent growth models with 2 slopes, regressed on study condition), but with norms and protective behavioral strategies serving as the outcomes of interest rather than alcohol use or related problems (for addressing primary aim 2). All models will be conducted in Mplus (version 8; Mplus) [71] using maximum likelihood estimation. These analyses assume normally distributed outcomes; thus, for any outcome that demonstrates nonnormality, competing approaches will be explored (eg, variable transformation vs specifying a different distribution), and we will choose the best-fitting model for the data.

For the exploratory secondary aim, R-Simulation Investigation for Empirical Network Analysis will be used to conduct stochastic, actor-based models [72]. These will allow us to examine whether behavior change precedes network changes (ie, selection) or whether network changes precede behavior change (ie, socialization). These models will be conducted only if reductions in drinking or related problems are observed in the primary aim 1 examination (ie, there is behavior change over time) and if several members of each organization participate (so that network change can be examined over time).

### Power Analysis

Power estimations were conducted using Monte Carlo simulation methods within a structural equation modeling framework [73]. Estimates of effect sizes, variances, and covariances were based on data from a preliminary study using a similar protocol (some participants received only the intervention, whereas others received the intervention and booster) among college drinkers [43]. A meta-analysis of randomized controlled trials assessing alcohol interventions for first-year college students indicated an average retention rate of 76% across studies [74]; therefore, this retention estimate was used for the power analysis. Monte Carlo simulation methods indicated that for the expected effect size (b=6.57; β=.537) and expected 24% attrition, total sample size of 180 students should yield power=0.82 to detect differences in the slope estimate across study conditions.

## Results

The IRB approval was obtained in November 2021, with the amendment to switch data collection sites approved in February 2022. Approval was obtained from the research committee of the National Panhellenic conference in January 2022. As of September 2022, we have enrolled 18 participants from 2 fraternities and 2 sororities, and they have completed the baseline survey. Of the 18 participants, 13 (72%) participants have completed the 1-month follow-up. We expect to complete enrollment by the end of 2022. Analysis has not yet begun, but is expected to begin immediately following the completion of the last follow-up assessment (ie, July 2023).

## Discussion

### Overview

This study addresses alcohol use and related problems among members of sororities and fraternities, an at-risk population that often engages in heavier drinking than their non-Greek counterparts, typically reporting more consequences [9,11-13]. As members of Greek life are a group that has not responded to previous intervention efforts [20], they are in need of efforts to strengthen and extend intervention effects. Personalized feedback boosters sent via email have led to further drinking reductions among select college drinkers [43,49], suggesting that they may be a promising tool for reducing drinking among members of fraternities and sororities. This study examines the efficacy of personalized boosters emailed after interventions to address alcohol use and related problems (primary aim 1) and normative perceptions and protective behavioral strategies (primary aim 2). We hypothesize initial postintervention drinking reductions for both study conditions, with individuals in the booster condition reporting further reductions at later follow-ups. We also hypothesize that individuals in the booster condition will report further reductions in norms and increases in protective behavioral strategies at later follow-ups.

Given the close connections among members of fraternities and sororities, these may be closed peer networks that can facilitate the examination of how changes in drinking occur through social influence. Thus, this study has an exploratory secondary aim to examine social mechanisms of change (ie, through selection vs socialization). This aim will be examined only if the booster is efficacious and if several members of each organization participate.

### Methodological Challenges and Consideration

This study has presented several unexpected challenges, leading to revisions in the protocol. After learning from the study advisory panel about the nature of Greek life at the host institution, a new data collection site was identified. Campus administrators also requested additional approvals beyond those required by federal guidelines. Finally, the data collection institution changed the programs addressing college drinking among their student body. To control for previous exposure to other programs, the project staff examined the new program and how comparable it was with the old program. Each of these challenges caused delay of a month or more, and collectively, they had a major impact on the study time line and therefore the study budget. By the time data collection was launched, the institution had begun its spring break. After returning from the spring break, students were focused on the final exams and end of the semester, thus hindering data collection.

Another major challenge is our attempt to recruit whole networks, because of which we cannot use typical data collection methods such as sending emails to the entire student body, posting student announcements, hanging flyers around the campus, and so on. To try to enroll entire organizations, we focus our recruitment efforts on chapter presidents, and then solicit their help with recruitment of their members (obtain member lists and email addresses, help in selecting promotional item, obtain access to chapter meetings for recruitment purposes, etc). This means that the president serves as a gatekeeper to their organization, and if they are not interested, are wary of unsolicited email, or do not keep up with their email, it prevents their entire organization from participating. We send recruitment emails from members of the research team both within and outside the data collection institution and at different times of the day to increase the probability of having any individual chapter president read the recruitment emails. This challenge was compounded by an institutional transition to a new student email provider, which resulted in a change in student email addresses. This occurred midyear, and there was confusion among students, who may not have been good at monitoring the new email address during this time.

Providing adequate compensation was also a challenge. As we are recruiting full networks, we needed to not only consider compensation for the individual participants but also for the group as a whole to incentivize participation. Owing to concerns of coercion or peer pressure (ie, providing an incentive if a specific percentage of the organization participated in the baseline survey), the compensation method suggested by the study advisory board was not approved.

### Institutional Administration

This study relies strongly on institutional administration to facilitate the study protocol (ie, providing information about existing alcohol harm reduction programs and connecting project staff with chapter presidents of fraternities and sororities). At the home institution, we have strong ties with the administrators who provide critical help to facilitate studies in launching quickly and running smoothly. These include individuals at the highest levels who strongly believe in the purpose of our research to help college students make healthy, responsible choices. It would have been beneficial to cultivate these connections at the data collection site also. When we had to change the recruitment site, we directly contacted the offices that can provide the information we needed and did not engage the high-level administrators. Although we provided ample information about the study purpose and the steps taken to secure participant confidentiality, we experienced delays and additional approval requirements. The process may have been fast and smooth if we had first cultivated a relationship with high-positioned administration officials who could have made these requests on our behalf, possibly with better results.

### Fully Remote Data Collection

We planned for fully remote data collection because of pandemic-related restrictions on in-person data collection at the host institution. Although these restrictions were being eased as we prepared to launch the study, we worried that they could be reintroduced if case counts rise. This was helpful, as we changed to data collection at another institution. The institution is nearby, but still approximately an hour away; we did not want this travel time as a requirement for the participants or research assistants. Remote data collection has been both an advantage and disadvantage. The student advisory panel indicated a strong preference for remote data collection via virtual meetings, rather than face-to-face meetings. This may be much more convenient for participants, as they do not have to travel to a specific location (or allow for travel time in their schedule). Volunteer research assistants have shared that they appreciate the convenience of this data collection method, and it allows for web-based students to obtain research experience, which is often a challenge. However, enrollment within an organization might be high if we could attend a chapter meeting in person with laptops or other devices to facilitate baseline session participation. It also presented some early challenges in terms of identifying a web-based scheduling system, creating meetings that will allow research assistants from different institutions to be the host, and so on, but all these challenges were resolved fairly easily.

### Informational Videos

Given the complexities of this study, we thought that it was of critical importance for participants to understand the protocol. Moreover, we have a large team of research assistants who are ready to help with data collection. As such, we created informational videos that provide all the information they need for the study (in addition to reading the informed consent document). The videos are created in a program that uses clip art–style images, allowing race, gender, and other identities to not be present in the videos, unlike if the researchers created videos of themselves presenting the information. Overall, 3 videos were created. The first video reviewed the purpose of the study and provided information about confidentiality to participants. The second video explained the steps involved in the baseline session and related compensation. The third video provided information about the long-term steps of the study (follow-up surveys and feedback emails) and related compensation. Using the videos to provide this information, rather than a research assistant, allows for participants who are late to the session to still receive the necessary information, without the research assistant needing to repeat themselves or distract participants who already began the survey (ie, lets participants go through the information at their own pace). In addition, it allows for a standardized way for participants to receive important information about the study. In other words, everyone hears and reads the same information, regardless of who is leading the session that day, thus providing guaranteed fidelity.

### Recommendations and Future Directions

Given the challenges we experienced, we list several recommendations for researchers. Cultivating a relationship with high-ranking administration officials can potentially help future researchers to either prevent or better address potential challenges. We may not have been required to secure additional approvals, may have received our requested information fast, and may have learned about upcoming changes early (for email and alcohol programming transitions). Our student advisory panel was incredibly helpful in forming the design of the study, and we recommend their use to other researchers moving into a new method or population. We recommend against study protocols that include single gatekeepers (in our case, chapter presidents); we suggest identifying other methods when possible. If gatekeepers are required, better methods should be identified to secure buy-in and promote group participation (such as our prohibited charity donation). We also recommend being proactive in addressing data quality, such as informational videos to ensure fidelity of information transfer to participants, and methods to detect survey inattention that can provide live feedback, such as attention check items.

### Study Implications and Potential Impact

Project Greek assesses the utility of an email-delivered personalized feedback booster after a web-based intervention with Greek-involved college students. This population typically drinks more heavily than their peers and is often resistant to intervention, making them an ideal population for this approach. Alcohol eCHECKUP TO GO was selected as the intervention owing to its empirical support and low cost. In addition, both the booster and intervention use mobile technology that allows for remote delivery. The results of this study may reveal a path for reducing drinking in this high-risk group, which does not require many resources and is both cost-effective and easy to disseminate. In summary, Project Greek will provide information about the efficacy of personalized feedback boosters after a web-based intervention to address risky drinking among members of Greek life. This is a promising avenue of research toward the goal of helping college students to make healthy, responsible choices about their drinking.

### Conclusions

Project Greek has great potential to address the high-risk drinking patterns documented repeatedly among members of Greek life. The protocol described here assesses the utility of personalized normative feedback delivered via email after a web-based intervention for members of fraternities and sororities, using the same automated technology and remote delivery as the original intervention. However, there were numerous challenges in launching the study, resulting in several updates to the protocol. These included changing the data collection institution and securing additional approvals to work with administrative offices on campus, among others. Persistent limitations include the use of a recruitment method that includes gatekeepers for access to other participants (ie, chapter presidents) and the inability to provide group-level incentives. Using a student advisory panel was a helpful approach for obtaining feedback about the protocol before launching it, and we recommend its use to other researchers.

## Appendix

Multimedia Appendix 1 Peer review report by Epidemiology, Prevention and Behavior Research Review Subcommittee - National Institute on Alcohol Abuse and Alcoholism (AA-2) Initial Review Group - National Institute on Alcohol Abuse and Alcoholism (National Institutes of Health, USA).

## Abbreviations

IRB: institutional review board

## Data Availability

Data will be available from the principal investigator upon reasonable request.
