# Supplementary material for: Examining Emailed Feedback as Boosters After a College Drinking Intervention Among Fraternities and Sororities: Rationale and Protocol for a Remote Controlled Trial (Project Greek)
Source: JMIR Res Protoc. 2022 Oct 28;11(10):e42535. doi: 10.2196/42535 (PMC9652738; doi:10.2196/42535)
Supplement: Multimedia Appendix 1 [file resprot_v11i10e42535_app1.pdf]

SUMMARY STATEMENT
(Privileged Communication)

PROGRAM CONTACT:
MARIELA SHIRLEY
301-443-9389
shirleym@mail.nih.gov

Release Date: **06/17/2015**
Revised Date: **07/15/2015**

Application Number: **1 K01 AA023849-01A1**

Principal Investigator

BRAITMAN, ABBY L. PHD

Applicant Organization: OLD DOMINION UNIVERSITY

Review Group: **AA-2**
Epidemiology, Prevention and Behavior Research Review Subcommittee

Meeting Date: **06/01/2015** ***RFA/PA:*** **PA14-044**
Council: **OCT 2015** ***PCC:*** **AP C**
Requested Start: **09/01/2015**

Project Title: **Refining Boosters to Strengthen Online College Student Drinking Interventions**

SRG Action: **Impact Score: 20**
Next Steps: **Visit http://grants.nih.gov/grants/next_steps.htm**
Human Subjects: 30-Human subjects involved - Certified, no SRG concerns
Animal Subjects: 10-No live vertebrate animals involved for competing appl.
Gender: **1A-Both genders, scientifically acceptable**
Minority: **1A-Minorities and non-minorities, scientifically acceptable**
Children: **1A-Both Children and Adults, scientifically acceptable**
Clinical Research - not NIH-defined Phase III Trial

Project Year	Direct Costs Requested	Estimated Total Cost
1	129,863	140,252
2	123,493	133,372
3	124,452	134,408
4	141,016	152,297
5	125,367	135,396
TOTAL	644,191	695,725

ADMINISTRATIVE BUDGET NOTE: The budget shown is the requested budget and has not been adjusted to reflect any recommendations made by reviewers. If an award is planned, the costs will be calculated by Institute grants management staff based on the recommendations outlined below in the COMMITTEE BUDGET RECOMMENDATIONS section.

1K01AA023849-01A1 Braitman, Abby

REVISED

RESUME AND SUMMARY OF DISCUSSION: This amended K01 grant application is proposing to expand the candidate's knowledge of college drinking to include social network analysis and evaluation of booster interventions for college drinkers. The training plan includes workshops, professional meetings, guided readings and meetings with mentors. The research plan is proposing to modify an email booster program for a brief online alcohol use prevention intervention for college students. The candidate is extremely productive; she has 13 publications. The application is responsive to the previous concerns. For example, a mentor with social network analysis research expertise has been added to the mentoring team, additional social network analysis training opportunities were added, the research plan was revised and the candidate explained what skills she would gain from this opportunity. There is a high level of enthusiasm for this candidate rated in the exceptional-outstanding range.

DESCRIPTION (provided by applicant): This application is for a Mentored Research Scientist Development Award (MRSDA; K01). Abby L. Braitman, Ph.D., is an excellent candidate for this award. Her burgeoning program of research focuses broadly on harm reduction for emerging adult drinkers, with a specific focus on boosters to strengthen and extend the effects of popular online interventions for college drinking. Dr. Braitman [was previously] a National Research Service Award (NRSA) postdoctoral fellow at Old Dominion University, and the MRSDA would facilitate her goals of becoming a productive, independent scientist researching the reduction of alcohol-related problems among emerging adults. To strengthen her current skill set, broaden her knowledge to a wider scope, and add new skills to her repertoire, the following training goals have been identified for the award period: 1) to broaden and strengthen her emerging adult alcohol use knowledge base, 2) to develop expertise in the enhancement and evaluation of boosters to interventions for college drinkers, 3) to increase her proficiency in the principles and skills of Social Network Analysis, 4) to enhance scholarly productivity via secondary data analyses and manuscript preparation, and 5) to increase her skills and productivity regarding grantsmanship. The training and research plan enlists the mentorship and collaboration of Kate Carey, Ph.D., Professor of Behavioral & Social Sciences at Brown University, Michelle Kelley, Ph.D., Professor of Psychology, and Cathy Lau-Barraco, Ph.D., Assistant Professor of Psychology, both at Old Dominion University, [as well as Kayo Fujimoto, Ph.D., Assistant Professor in the School of Public Health at the University of Texas], with the consultant Ken Leonard, Ph.D., Senior Research Scientist and Director of the Research Institute on Addictions (RIA) out of SUNY, Buffalo. Old Dominion University, a large, state-supported institution in Norfolk, Virginia is an ideal institutional environment to carry out these goals, with numerous resources in support of both the training and research goals specified. The research activities under the proposed award include a series of three studies designed to refine the methodology, improve the efficacy of an electronic booster, [and close the gap between in-person and computerized interventions]; this program of research aims to 1) examine if personal contact enhances the tailored feedback received via booster email, 2) identify optimal timing of delivery for booster, [3a) explore social mechanisms of change], 3b) examine the effects of interventions-plus-boosters when delivered to existing social networks (i.e., fraternities/sororities, and individual drinking networks), and 4) examine previously identified potential moderators and mediators of reductions in alcohol use and related problems. Results from the proposed research will provide preliminary data for an R01 application to be completed by Dr. Braitman during the award period. Completion of the proposed training and research would allow her to transition to independence as an investigator and lay the groundwork for her long-term career goals.

PUBLIC HEALTH RELEVANCE

The goal of the proposed research is to streamline and improve the efficacy of an innovative booster designed to strengthen and extend the effects of a computerized intervention targeting college drinking.

The booster is low-cost and easy to disseminate, thus implementable on a large scale. Consequently, the public health impact may be far-reaching.

CRITIQUE 1:

Candidate: 1
Career Development Plan/Career Goals /Plan to Provide Mentoring: 2
Research Plan: 3
Mentor(s), Co-Mentor(s), Consultant(s), Collaborator(s): 1
Environment Commitment to the Candidate: 1

Overall Impact:

This K01 application proposes to refine a recently-developed (by the applicant) email booster program for a brief online alcohol use prevention intervention for young adult college students. Training goals include furthering expertise in emerging adult alcohol use and evaluation of booster sessions and to train in social network analysis. The applicant is promising with a clear program of research and the mentoring team is very good.

The applicant was responsive to most of the critiques. Most notably, the mentoring team now includes an expert on social network analyses, and the social network analysis section of the training plan and the research study is now more well-developed.

There are a few lingering concerns regarding further development of SNA analysis and greater explanation given to the *new* benefits of this training plan, but otherwise this is a very good application and much improved.

1. Candidate:

Strengths

- Solid publication record with 13 pubs, several first authored and in her line of research.
- Applicant has an F32 postdoctoral award from NIAAA.

Weaknesses

- None noted

2. Career Development Plan/Career Goals & Objectives/Plan to Provide Mentoring:

Strengths

- The applicant's training plan is detailed and feasible.
- The training (including new training opportunities added in this revision) in social network analysis is a strength.

Weaknesses

- The applicant somewhat clarified what new skills would be learned from the first goal of training in young adult alcohol use (by stating that she would learn about mobile technology and tracking of alcohol use), but that new skill is not well-integrated into the research plan and into her future career trajectory.

3. Research Plan:

Strengths

- Booster sessions as supplements to online interventions have the promise of bridging the gap in effect sizes from online and in-person interventions.
- Social network analyses of *how* online interventions and boosters work (through socialization and selection effects within networks) is novel and is now better developed.
- The two adjustments to the booster sessions (personalized booster contact and timing of boosters) that will be tested are now clearly justified based on the literature.
- Research plan follows clearly from applicant's program of research.

Weaknesses

- It is stated that study 3 will examine the impact of delivery of the intervention and booster sessions to a social network (fraternities and sororities) on intervention efficacy. BUT, Study 3 examines an intervention (with or without booster) delivered to students recruited through fraternities and sororities. All subjects get the intervention. Thus, it is unclear how this study can test whether the intervention is more effective being delivered within social networks vs. to individuals since there was not random assignment of delivery of the intervention.
- The section on SNA analyses is much improved, but it still is a bit underdeveloped. For example, the analysis section did not detail how it would treat socialization effects for participants who had many friends who did the intervention vs. those who only had a few friends (or, for example, sorority members) who consented to participate in the intervention.

4. Mentor(s), Co-Mentor(s), Consultant(s), Collaborator(s):

Strengths

- Primary sponsor is now Carey, who has expertise in booster sessions.
- Second sponsor Kelly has expertise in youth drinking and harm reduction and in interventions and is PI on one R01 through NIDA.
- Added a mentor in SNA analyses.

Weaknesses

- Given that Dr. Kelly was the mentor for the applicant's F32 fellowship, the applicant changed the primary sponsor to be Carey. That makes sense, but it would be good to outline the novel training opportunities that Carey would provide in a bit more detail.

5. Environment and Institutional Commitment to the Candidate:

Strengths

- Solid research environment with good facilities

Weaknesses

- None noted.

Protections for Human Subjects:

Acceptable Risks and Adequate Protections

Data and Safety Monitoring Plan (Applicable for Clinical Trials Only):

Acceptable

Inclusion of Women, Minorities and Children:

G1A - Both Genders, Acceptable

M1A - Minority and Non-minority, Acceptable

C1A - Children and Adults, Acceptable

- Acceptable.

Training in the Responsible Conduct of Research:

Acceptable

Comments on Format (Required):

- Good- a mix of workshops at ODU and at conferences and meetings with sponsors.

Comments on Subject Matter (Required):

- Subject matter stated- a wide range.

Comments on Faculty Participation (Required; not applicable for mid- and senior-career awards):

- Acceptable

Comments on Duration (Required):

- Throughout the award.

Comments on Frequency (Required):

- Frequency of workshops is now stated.

Resource Sharing Plans:

Acceptable

Budget and Period of Support:

Recommend as Requested

CRITIQUE 2:

Candidate: 1
Career Development Plan/Career Goals /Plan to Provide Mentoring: 2
Research Plan: 1
Mentor(s), Co-Mentor(s), Consultant(s), Collaborator(s): 1
Environment Commitment to the Candidate: 1

Overall Impact:

This K01 application proposes to expand the applicant's ability to become an independent researcher through examining various aspects of harm reduction for college student drinking. The proposed project is of value given the deteriorating effects of alcohol prevention/intervention initiatives for college students and the increased emphasis on computer based interventions even though in person interventions tend to outperform computer interventions. Thus, the research proposed attempts to bridge the gap that exists between these two interventions bu studying booster sessions and eventually the involvement of social networks. The training plan is solid and the mentoring team is strong

especially given the addition of a social network expert. The applicant has demonstrated strong promise to develop into an independent researcher. The only main concern that still exists is the limited exposure the applicant has had outside to research training outside of her doctoral training program and postdoc (all at ODU). Thus, her training has appears to be vary narrowed with this limited exposure.

1. Candidate:

Strengths

- The applicant has an impressive publication history which shows promise of further development as an independent researcher.
- The applicant has received a NIAAA F 32.

Weaknesses

- None noted

2. Career Development Plan/Career Goals & Objectives/Plan to Provide Mentoring:

Strengths

- The applicant’s plan is feasible and details the development of scientific ability.
- The coursework and interactions with mentors are strong.

Weaknesses

- It remains unclear how new skills will be developed through the first 2 goals and how this differentiates from the F 32. A great

3. Research Plan:

Strengths

- The research plan is consistent with the applicant’s research focus.
- It is important to better appreciate the value or lack of for booster sessions.
- Helping to understand the role of personal contact is very important.

Weaknesses

- None

4. Mentor(s), Co-Mentor(s), Consultant(s), Collaborator(s):

Strengths

- The mentoring team is strong with varied expertise and mentoring experience.
- The mentoring team was reorganized and additional expertise was added.

Weaknesses

- None noted

5. Environment and Institutional Commitment to the Candidate:

Strengths

- The environment and institutional commitment appear to be acceptable.

Weaknesses

- It is unclear why the applicant will need to purchase computers and software if those are already available – as indicated as already available to the applicant in the facilities?

Protections for Human Subjects:

Acceptable Risks and Adequate Protections

Data and Safety Monitoring Plan (Applicable for Clinical Trials Only):

Acceptable

Inclusion of Women, Minorities and Children:

G1A - Both Genders, Acceptable

M1A - Minority and Non-minority, Acceptable

C1A - Children and Adults, Acceptable

Training in the Responsible Conduct of Research:

Acceptable

Comments on Format (Required):

- Appropriate format

Comments on Subject Matter (Required):

- Covers a wide variety of topics

Comments on Faculty Participation (Required; not applicable for mid- and senior-career awards):

- Acceptable

Comments on Duration (Required):

- Acceptable - throughout funding years

Comments on Frequency (Required):

- Acceptable

Resource Sharing Plans:

Acceptable

Budget and Period of Support:

Recommend as Requested

CRITIQUE 3:

Candidate: 2
Career Development Plan/Career Goals /Plan to Provide Mentoring: 1

Research Plan: 2
Mentor(s), Co-Mentor(s), Consultant(s), Collaborator(s): 1
Environment Commitment to the Candidate: 1

Overall Impact:

This revised application by a highly qualified and promising applicant is very responsive to feedback from reviewers. The applicant has modified her mentoring team to include an expert on social network analysis and has redistributed the role of mentors to allow for novel mentoring experiences with an off-site mentor. She has strengthened her research plan and provided greater details on key training components. Overall, this is a strong application.

1. Candidate:

Strengths

- As before, an excellent candidate with a strong background in alcohol research.
- Prior recipient of a NIAAA F32.
- A couple of new pubs since last submission.

Weaknesses

- The prior submission listed 3 first authored papers under review but it is not indicated whether these are in press/under revision etc.

2. Career Development Plan/Career Goals & Objectives/Plan to Provide Mentoring:

Strengths

Weaknesses

- None

3. Research Plan:

Strengths

- 3 aims: to bridge gap between online and in-person interventions; timing of booster delivery (2-14 wks.); role of social networks in booster efficacy. Aims are much tighter in this revised version and more compelling.
- Major concern #1 on last submission was that evidence for effectiveness of booster was limited, except in applicant’s work. This is partially addressed by detailing differences in the applicant’s work relative to other research.
- Major concern #2 was lack of research on delivery of intervention via social networks which is problematic in the absence of random assignment of participants. Cluster-randomization is proposed.
- Major concern #3 was lack of details on the SNA. This has been carefully addressed (e.g. use of SAOMs).

Weaknesses

- A minor concern is the proposal to develop a competitive R01 in year 5. Given the current likelihoods of success with R01 submissions, relegating R01 submission to year 5 appears shortsighted.

- Aim 4 continues to be under-developed. For instance, C7 describes specific and detailed plans for Aims 1-3 but not aim 4.

4. Mentor(s), Co-Mentor(s), Consultant(s), Collaborator(s):

Strengths

- Mentors are well-suited to the project. Drs. Kelley, Carey and Lau-Barraco have published with the applicant.
- A concern with the prior submission was the prominent role of the F32 mentor, Dr. Kelley, in the proposal. This is ameliorated with the more prominent involvement of Dr. Carey, who despite being off-site, is forging new research pathways with the applicant.
- The addition of Dr. Fujimoto provides much needed expertise in SNA (however, please note that personal statement of Dr. Fujimoto was not edited to reflect role on this K01).
- Dr. Leonard continues to bring a unique perspective on the role of social/interpersonal relationships in alcohol use to the proposal.

Weaknesses

- None

5. Environment and Institutional Commitment to the Candidate:

Strengths

- Excellent resources and institutional commitment

Weaknesses

- None

Protections for Human Subjects:

Acceptable Risks and Adequate Protections

Data and Safety Monitoring Plan (Applicable for Clinical Trials Only):

Inclusion of Women, Minorities and Children:

G1A - Both Genders, Acceptable

M1A - Minority and Non-minority, Acceptable

C1A - Children and Adults, Acceptable

Training in the Responsible Conduct of Research:

Acceptable

Comments on Format (Required):

- Acceptable (face-to-face)

Comments on Subject Matter (Required):

- Acceptable

Comments on Faculty Participation (Required; not applicable for mid- and senior-career awards):

- Yes

Comments on Duration (Required):

- Annual

Comments on Frequency (Required):

- Acceptable

Budget and Period of Support:

Recommend as Requested

THE FOLLOWING SECTIONS WERE PREPARED BY THE SCIENTIFIC REVIEW OFFICER TO SUMMARIZE THE OUTCOME OF DISCUSSIONS OF THE REVIEW COMMITTEE, OR REVIEWERS' WRITTEN CRITIQUES, ON THE FOLLOWING ISSUES:

THE FOLLOWING SECTIONS WERE PREPARED BY THE SCIENTIFIC REVIEW OFFICER TO SUMMARIZE THE OUTCOME OF DISCUSSIONS OF THE REVIEW COMMITTEE, OR REVIEWERS' WRITTEN CRITIQUES, ON THE FOLLOWING ISSUES:

PROTECTION OF HUMAN SUBJECTS (Resume): ACCEPTABLE

INCLUSION OF WOMEN PLAN (Resume): ACCEPTABLE

INCLUSION OF MINORITIES PLAN (Resume): ACCEPTABLE

INCLUSION OF CHILDREN PLAN (Resume): ACCEPTABLE

COMMITTEE BUDGET RECOMMENDATIONS: The budget was recommended as requested.

REVISION NOTE: The summary statement was revised and re-released on 7/15/2015.

NIH has modified its policy regarding the receipt of resubmissions (amended applications). See Guide Notice NOT-OD-14-074 at http://grants.nih.gov/grants/guide/notice-files/NOT-OD-14-074.html. The impact/priority score is calculated after discussion of an application by averaging the overall scores (1-9) given by all voting reviewers on the committee and multiplying by 10. The criterion scores are submitted prior to the meeting by the individual reviewers assigned to an application, and are not discussed specifically at the review meeting or calculated into the overall impact score. Some applications also receive a percentile ranking. For details on the review process, see http://grants.nih.gov/grants/peer_review_process.htm#scoring.

MEETING ROSTER

Epidemiology, Prevention and Behavior Research Review Subcommittee
National Institute on Alcohol Abuse and Alcoholism Initial Review Group
NATIONAL INSTITUTE ON ALCOHOL ABUSE AND ALCOHOLISM
AA-2 1
June 01, 2015

CHAIRPERSON

D'AMICO, ELIZABETH J, PHD
SENIOR BEHAVIORAL SCIENTIST
RAND CORPORATION
SANTA MONICA, CA 90407

MEMBERS

AGRAWAL, ARPANA , PHD
ASSOCIATE PROFESSOR
DEPARTMENT OF PSYCHIATRY
WASHINGTON UNIVERSITY IN ST. LOUIS
ST. LOUIS, MO 63110

CAPALDI, DEBORAH M, PHD
SENIOR SCIENTIST
OREGON SOCIAL LEARNING CENTER
EUGENE, OR 97405

CARPENTER, CHRISTOPHER , PHD *
PROFESSOR
DEPARTMENT OF ECONOMICS
VANDERBILT UNIVERSITY
NASHVILLE, TN 37235

CHAMBERS, CHRISTINA , PHD *
ASSOCIATE PROFESSOR
DEPARTMENT OF PEDIATRICS
UNIVERSITY OF CALIFORNIA, SAN DIEGO
LA JOLLA, CA 92093

CHAPLIN, TARA MARIE, PHD *
PRINCIPAL INVESTIGATOR
DEPARTMENT OF PSYCHOLOGY
GEORGE MASON UNIVERSITY
FAIRFAX, VA 22030

FELL, JAMES C, MS
SENIOR RESEARCH SCIENTIST
PACIFIC INSTITUTE FOR RESEARCH AND EVALUATION
CALVERTON, MD 20705

FIELD, CRAIG A, PHD *
ASSOCIATE PROFESSOR
UNIVERSITY OF TEXAS AT AUSTIN
CENTER FOR SOCIAL WORK RESEARC
HEALTH BEHAVIOR RESEARCH
AUSTIN, TX 78703

HAM, LINDSAY S., PHD *
ASSOCIATE PROFESSOR
DIRECTOR OF CLINICAL TRAINING
DEPARTMENT OF PSYCHOLOGICAL SCIENCES
UNIVERSITY OF ARKANSAS
FAYETTEVILLE, AR 72701

HETTEMA, JENNIFER ELIN, PHD *
ASSISTANT PROFESSOR
DEPARTMENT OF PSYCHIATRY & NEUROBEHAVIORAL SCIENCE
DIVISION OF NEUROBIOLOGICAL STUDIES
UNIVERSITY OF VIRGINIA
RICHMOND, VA 23294

LEEMAN, ROBERT F, PHD *
ASSISTANT PROFESSOR
DEPARTMENT OF PSYCHIATRY
YALE SCHOOL OF MEDICINE
NEW HAVEN, CT 06519

MADSON, MICHAEL BRIAN, PHD *
ASSOCIATE PROFESSOR
DEPARTMENT OF
UNIVERSITY OF SOUTHERN MISS
HATTIESBURG, MS 39406

MAGGS, JENNIFER L, PHD *
ASSOCIATE PROFESSOR
DEPARTMENT OF HUMAN DEVELOPMENT
AND FAMILY STUDIES
PENNSYLVANIA STATE UNIVERSITY
UNIVERSITY PARK, PA 168026504

MAHER, BRION S, PHD *
ASSOCIATE PROFESSOR
DEPARTMENT OF MENTAL HEALTH
JOHNS HOPKINS BLOOMBERG SCHOOL OF PUBLIC HEALTH
BALTIMORE, MD 21205

MCCARTHY, DENIS M, PHD
ASSOCIATE PROFESSOR
DEPARTMENT OF PSYCHOLOGICAL SCIENCES
UNIVERSITY OF MISSOURI
COLUMBIA, MO 65211

MCCAULEY OHANNESSIAN, CHRISTINE , PHD *
ASSOCIATE PROFESSOR
DEPARTMENT OF PEDIATRICS
UNIVERSITY OF CONNECTICUT MEDICAL SCHOOL
DIRECTOR, CHILDREN'S CENTER FOR COMMUNITY RESEARCH
CONNECTICUT CHILDREN'S MEDICAL CENTER
HARTFORD, CT 06106

MILLER, LESLIE M, PHD *
EXECUTIVE DIRECTOR
CENTER FOR TECHNOLOGY
IN TEACHING AND LEARNING
RICE UNIVERSITY
HOUSTON, TX 77005

MUN, EUN-YOUNG , PHD
ASSISTANT PROFESSOR OF PSYCHOLOGY
CENTER OF ALCOHOL STUDIES & GRADUATE SCHOOL OF APPLIED & PROFESSIONAL PSYCHOLOGY
RUTGERS, THE STATE UNIVERSITY OF NEW JERSEY
PISCATAWAY, NJ 08854

MUNDT, MARLON PAUL, PHD *
ASSISTANT PROFESSOR
HEALTH ECONOMIST
UNIVERSITY OF WISCONSIN-MADISON
MADISON, WI 53715

SAFFER, HENRY , PHD *
RESEARCH ASSOCIATE
NATIONAL BUREAU OF ECONOMIC RESEARCH
NEW YORK, NY 100164309

SMITH, GORDON STEPHEN , MD *
PROFESSOR
DEPARTMENT OF EPIDEMIOLOGY & PREVENTIVE MEDICINE
UNIVERSITY OF MARYLAND SCHOOL OF MEDICINE
BALTIMORE, MD 21201

TRIM, RYAN SCOTT, PHD *
PROFESSOR
DEPARTMENT OF MEDICINE
SCHOOL OF MEDICINE
UNIVERSITY OF CALIFORNIA-SAN DIEGO
SAN DIEGO, CA 92161

USDAN, STUART L, PHD *
ASSOCIATE PROFESSOR
DEPARTMENT OF HEALTH SCIENCE
UNIVERSITY OF ALABAMA
TUSCALOOSA, AL 35487-031

WALTERS, SCOTT T, PHD
PROFESSOR
BEHAVIORIAL AND COMMUNITY HEALTH
UNIVERSITY OF NORTH TEXAS SCHOOL OF PUBLIC HEALTH
FT. WORTH, TX 761072269

WILLIAMSON, DOUGLAS E, PHD
DIELMANN PROFESSOR OF GENETIC AND ENVIRONMENTAL RISK
DEPARTMENT OF PSYCHIATRY
UNIVERSITY OF TEXAS HEALTH SCIENCE CENTER
SAN ANTONIO, TX 78229

SCIENTIFIC REVIEW OFFICER

FOSTER, KATRINA L, PHD
SCIENTIFIC REVIEW ADMINISTRATOR
EXTRAMURAL PROJECT REVIEW BRANCH
OFFICE OF EXTRAMURAL ACTIVITIES
NATIONAL INSTITUTE ON ALCOHOL ABUSE AND ALCOHOLISM
NATIONAL INSTITUTES OF HEALTH
BETHESDA, MD 208929304

EXTRAMURAL SUPPORT ASSISTANT

LYLES, DEIRDRE
EXTRAMURAL SUPPORT ASSISTANT
NATIONAL INSTITUTES OF HEALTH
NATIONAL INSTITUTE OF ALCOHOL ABUSE AND ALCOHOLISM
OFFICE OF EXTRAMURAL ACTIVITIES
EXTRAMURAL PROJECT REVIEW BRANCH
BETHESDA, MD 208929304

* Temporary Member. For grant applications, temporary members may participate in the entire meeting or may review only selected applications as needed.

Consultants are required to absent themselves from the room during the review of any application if their presence would constitute or appear to constitute a conflict of interest.
